# Supplementary material for: Extracellular Vesicles from Uterine Aspirates Represent a Promising Source for Screening Markers of Gynecologic Cancers
Source: Cells. 2022 Mar 22;11(7):1064. doi: 10.3390/cells11071064 (PMC8997481; doi:10.3390/cells11071064)
Supplement: Supplementary file 1 [file cells-11-01064-s001.zip › cells-1639720-supplementary.pdf]

cells MDPI

Supplementary

Table S1. Sequences of reverse transcription primers used; sequences of RT-qPCR primers and TaqMan™ probes used.

	Target	Sequence 5′-3′
Reverse Transcription primers	miR-199a-3p RT	GTCGTATCCAGTGCAGGGTCCGAGGTATTCGCACTGGATACGAC taacca
	miR-375-3p RT	GTCGTATCCAGTGCAGGGTCCGAGGTATTCGCACTGGATACGAC tcacgc
	miR-451a RT	GTCGTATCCAGTGCAGGGTCCGAGGTATTCGCACTGGATACGAC aactcag
	miR-23a-3p RT	GTCGTATCCAGTGCAGGGTCCGAGGTATTCGCACTGGATACGAC ggaaatc
	let-7b-5p RT	CTCAACTGGAGCTAGTTTCGTCGTAGGGCAGTTGAGAACCACAC
	miR-16-5p	GTTGGCTCTGGTGCAGGGTCCGAGGTATTCGCACCAGAGCCAAC cgccaa
PCR Reverse primer	Uni-Ch-R	GTGCAGGGTCCGAGGT
	R1 (only for let-7b)	CTGGAGCTAGTTTCGTCGTAG
PCR Forward primers	miR-199a-3p F	CAGCTGGGACAGTAGTCTGC
	miR-375-3p F	CACGCATAAACAAGCAAGC
	miR-451a F	CACGCATAAACCGTTACCA
	miR-23a-3p F	ATCACATTGCCAGGGATT
	let-7b-5p F	CAGCTGGGTGAGGTAGTAG
	miR-16-5p F	GTTTGGTAGCAGCACGTAAATA
PCR TaqMan probes with LNA modifications	miR-199a-3p P	**FAM**-CATTGGTTAGTCGTATC **-BHQ1**
	miR-375-3p P	**FAM**-CTCGCGTGAGTCGTATCC**-BHQ1**
	miR-451a P	**FAM**-TTACTGAGTTGTCGTATCC-**BHQ1**
	miR-23a-3p P	**FAM**-CGCACTGGATACGACGGAAATCC-**BHQ1**
	let-7b-5p P	**HEX-**GTTGTGTGGTTCTCAACTG-**BHQ1**
	miR-16-5p P	**FAM**-TTGGCGGTTGGCTCTG-**BHQ1**

Table S2. Ct values and standard deviation of hsa-miR-451a, hsa-miR-199a-3p, hsa-miR-375-3p, hsa-miR23a-3p, hsa-miR16-5p, let-7b-5p levels obtained by RT-qPCR in EVs isolated from UA of EOC patients and healthy individuals.

	**hsa-miR451a**		**hsa-miR375-3p**		**hsa-miR199a-3p**		**hsa-miR23a-3p**		**hsa-miR16-5p**		**let-7b-5p**	
Sample	mean Ct	SD	mean Ct	SD	mean Ct	SD	mean Ct	SD	mean Ct	SD	mean Ct	SD
N1	29.55	0.15	36.53	0.49	28.29	0.05	26.55	0.09	33.03	0.29	29.77	0.24
N2	28.51	0.13	36.38	0.26	26.60	0.34	25.63	0.17	31.57	0.30	27.29	0.16
N3	28.88	0.07	35.12	0.06	28.40	0.07	25.42	0.27	30.38	0.25	30.25	0.04
N4	26.82	0.07	37.11	0.33	27.53	0.04	25.01	0.08	27.88	0.16	27.97	0.09
N5	28.49	0.05	34.44	0.13	28.33	0.06	25.17	0.12	29.99	0.15	28.30	0.19
T1	30.09	0.04	34.15	0.11	29.86	0.08	25.50	0.09	24.09	0.09	28.47	0.07
T2	28.67	0.05	35.58	0.34	30.49	0.07	25.27	0.21	30.65	0.24	29.62	0.10
T3	29.44	0.13	34.88	0.09	31.80	0.07	25.99	0.05	35.28	0.27	27.935	0.16
T4	33.10	0.08	34.15	0.04	33.50	0.03	26.45	0.02	31.77	0.17	31.11	0.24
T5	31.92	0.31	33.64	0.11	29.91	0.10	25.64	0.09	31.54	0.33	25.67	0.04
